# Exploring affinity between organic probes and Prussian Blue Analogues via inverse gas chromatography

**DOI:** 10.1038/s41598-024-62939-7

**Published:** 2024-06-17

**Authors:** Stijn Paulusma, Kaustub Singh, Tom Smeding, Jayaruwan G. Gamaethiralalage, Frank W. Claassen, Hans Beijleveld, Hans-Gerd Janssen, Louis C. P. M. de Smet

**Affiliations:** 1 Laboratory of Organic Chemistry, Wageningen University & Research, Stippeneng 4, 6708 WE Wageningen, The Netherlands; 2 Department of Chemical Engineering, Delft University of Technology, Van Der Maasweg 9, 2628 CN Delft, The Netherlands; 3 Section of Chemical Science and Engineering, Department of Chemistry and Bioscience, Aalborg University, Niels Bohrs Vej 8, 6700 Esbjerg, Denmark; 4 Unilever Foods Innovation Centre-Hive, Bronland 14, 6708 WH Wageningen, The Netherlands; 5 Present Address: FrieslandCampina Innovative Centre, Bronland 20, 6708 WH Wageningen, The Netherlands

**Keywords:** Chemistry, Analytical chemistry, Materials chemistry, Physical chemistry

## Abstract

Prussian Blue Analogues (PBAs), which are characterized by their open structure, high stability, and non-toxic properties, have recently been the subject of research for various applications, including their use as electrode precursors for capacitive deionization, gas storage, and environmental purification. These materials can be readily tailored to enhance their affinity towards gases for integration with sensing devices. An improved understanding of PBA-gas interactions is expected to enhance material development and existing sensor deposition schemes greatly. The use of inverse gas chromatography (IGC) is a robust approach for examining the relationship between porous materials and gases. In this study, the adsorption properties of (functionalized) hydrocarbons, i.e., probe molecules, on the copper hexacyanoferrate (CuHCF) lattice were studied via IGC, demonstrating that alkylbenzenes have a higher affinity for this material than *n*-alkanes. This difference was rationalized by steric hindrance, π–π interactions, and vapour pressure effects. Along the same line, the five isomers of hexane showed decreasing selectivity upon increased steric hindrance. Enthalpy values for *n*-pentane, *n*-hexane and *n*-heptane were lower than that of toluene. The introduction of increased probe masses resulted in a surface coverage of 46% for toluene. For all *n*-alkane probe molecules this percentage was lower. However, the isotherms of these probes did not show saturation points and the observed linear regime proves beneficial for gas sensing. Our work demonstrates the versatility of CuHCF for gas sensing purposes and the potential of IGC to characterize the adsorption characteristics of such a porous nanomaterial.

## Introduction

Gas sensing is a multi-billion euro market and relevant for diverse areas, including environmental^1^, food safety^2^, medical monitoring^3^, security applications^4^, and various industrial processes^5^. Currently, most gas sensors are based on metal oxides^6,7^, which typically require high operation temperatures to adsorb gases (> 200 °C), and often show a significant cross-sensitivity. Novel strategies that enable room temperature (RT) operation^8,9^ include the utilization of advanced sensing schemes in metal oxide semiconductor sensors^10,11^ and conductive polymers in resistive sensors^12^. Porous nanomaterials hold promise for RT sensing applications and allow tunable selectivity given the latest developments in their design and synthesis and their excellent performance in gas separation studies^13–16^. While porous nanomaterials with luminescent properties have been explored for sensing schemes based on optical read-outs^11^, their use in electrical sensors intrinsically has a far wider applicability^17,18^. This field is less mature and requires further development of advanced deposition schemes to integrate porous nanomaterials with sensor platforms.

Over the last three decades, the variety of functional porous nanomaterials has been growing rapidly^16,19^. One such class of porous nanomaterials is the one of coordination polymers (CPs), consisting of metal ions and organic ligands interconnected via coordination bonds^20^. Their adjustable porosity and tunable functionalization through the choice of metal and organic ligands, makes them attractive for various applications^20–22^. Metal–organic Frameworks (MOFs) represent a major category of coordination polymers, which includes Prussian Blue Analogues (PBAs) as a distinctive subclass^23^. While MOFs have been extensively studied in the fields of catalysis and gas detection across various target analytes, particularly under RT conditions^24,25^, most PBA studies investigate their redox properties^26^ within the fields of energy storage, electrocatalysis, and capacitive deionization^27,28^. Given their facile, scalable synthesis as well as their ease of integration with sensing devices^23,29^, the potential of PBAs for the purpose of gas sensing is far reaching. A comprehensive understanding of the physicochemical properties of PBAs would further explore this potential, advancing their use in gas sensing^30^.

When screening nanomaterials like PBAs for their desired gas sensing purposes, and understanding material-gas interactions, additional analytical techniques can be beneficial. Once such technique is inverse gas chromatography (IGC). In the 1960s, Kiselev coined this term, where ‘inverse’ relates to the purpose of the experiment, i.e., the examination of the stationary phase properties rather than those of the (gaseous) compounds that are being separated. In IGC, a vaporized target analyte (hereafter referred to as probe or probe molecule) passes through a column containing the solid material being examined, generating a variety of chromatographic parameters such as retention times and the shape of chromatographic peaks^31,32^. Hence, IGC can provide valuable information on specific and dispersive interaction energies between the probe and the column material.

While porous nanomaterials such as MOFs^33,34^ and imine-based 2D COFs^33,34^, have been characterized extensively using IGC, only a few studies report its use for quantitative analysis of PBA-gas interactions, in particular the interactions with hydrocarbons. Volatile hydrocarbons, like short-chain-length alkanes and low-molecular-weight aromatic derivatives, are of particular interest given their role in fields like the oil and gas industry^35,36^, and environmental pollution^36–38^. Within the context of PBA, Autie-Castro et al. studied the adsorption and separation of propane and propylene on cobalt-based hexacyanometallates, Co_3_[Co(CN)_6_]_2_ and Zn_3_[Co(CN)_6_]^39^, while in a subsequent study IGC was utilized to evaluate the acid–base characteristics of these two PBAs with several probes, including several *n*-alkanes^40^.

While the incorporation of Co and/or Zn resembles only two analogues, a wide variety of transition metals can be used to make various PBAs. As such, a wide array of customizations in terms of the lattice is readily available^41^, making PBAs highly attractive for gas sensing applications^23,42,43^. Within the vast family of PBAs, we start with copper hexacyanoferrate (CuHCF)^44^ as it has been shown to be able to host mono, di as well as trivalent ions in its lattice^45^, making it ideal as a starting point for affinity-based applications. Furthermore, they can be synthesized with ease and also in combination with other transition metals such as nickel to give a mixed metal lattice like CuNiHCF^46^.

Here, we studied the interactions between CuHCF and alkanes, as well as aromatic alkylbenzenes, and polar probes. Then we determined the selectivity of hexane and its isomers towards CuHCF by comparing their retention times in IGC. Next, we obtained the adsorption enthalpies for selected probe molecules by assessing the temperature dependence of the retention volumes. Finally, we quantified the adsorption capacity of the selected hydrocarbons by injecting increased probe masses and evaluating the difference between the aromatic and linear ones.

## Experimental section

### Materials

CuCl_2_ (99%) and Na_4_Fe(CN)_6_ (99%) were purchased from Acros Organics and Sigma Aldrich, respectively. Methane (99%) and *n*-propane (99%) were purchased from SOL, ethane (99%) from Messer, *n*-butane (80%) from Campingaz, *n*-pentane (99%) from Merck, *n*-hexane (99%), *n*-decane (99%) and toluene (99%) from Honeywell. *N*-heptane (99%), *n*-octane (98%), *n*-nonane (99%), *n*-propylamine (99%), *n*-butylamine (99%), triethylamine (99%), aniline (99%), dimethylaniline (99%) and 2-methylpropa-2-ol (99%) were purchased from Sigma Aldrich and *n*-dodecane (99%) from Acros Organics. Ethylbenzene (99.8%), *n*-propylbenzene (97.5%), *n*-butylbenzene (99%), *n*-pentylbenzene (96%), *n*-hexylbenzene (98%,) and *n*-hexylamine (99%) were purchased from Thermo Scientific. Methylamine (40% in H_2_O), ethylamine (70% in H_2_O) and *n*-pentylamine (99%) were purchased from TCI chemicals. All chemicals were used as received.

### PBA synthesis and characterization

CuHCF was synthesized via a co-precipitation method according to a previous protocol by Singh et al.^47^ In short, 500 mL of an aqueous solution of 36 mM of CuCl_2_ and 18 mM of Na_4_Fe(CN)_6_ each was added drop-wise to a 1.5 L beaker containing 500 mL of MQ H_2_O. A Cole-Parmer Masterflex L/S peristaltic pump (Vernon Hills, United States) was used to add the copper and ferrocyanide solutions dropwise (5 mL/min) into the large beaker while stirring the solution at 1000 rpm. The product was filtered via Büchner filtration and dried in a vacuum oven at 120 °C to yield 10.1 g powder. This powder was milled (400 RPM, 1 min milling followed by 1 min pause for four consecutive cycles) with a Frisch Pulverisette 5 Premium line (Ede, The Netherlands), aiming to reduce the particle size and increase the homogeneity.

The crystallinity of the resulting milled CuHCF particles was assessed via powder X-ray diffraction (XRD) with diffraction angles between 5 and 90° with a Philips X’pert-PRO (Almelo, The Netherlands) at 40 kV and 40 mA. XRD samples were dried at 333, 423 and 473 K, respectively. Next, the milled powder particle distribution was determined via Scanning Electron Microscopy (SEM). CuHCF particles were gold coated to enhance resolution, prior to SEM imaging using a JEOL JFC-1300 coater (Tokyo, Japan), and imaged at increasing levels of magnification. The thermal stability was assessed via thermal gravimetric analysis (TGA) on an STA 6000 instrument (Zwijndrecht, The Netherlands) under a continuous N_2_ stream at a temperature range of 298-573 K.

### IGC equipment and column packing

IGC experiments were carried out using a modified Agilent 6850 series II Gas chromatograph (Amstelveen, The Netherlands) equipped with an FID detector, a split injector, and a transfer capillary (Fig. S6C). Glass tubes with dimensions of 78 × 4 mm (length × internal diameter) were used to prepare IGC columns (Figs. S7A,B). Briefly, one side of the tube was closed with dimethyldichlorosilane (DMCS)-treated glass wool, before packing it with 0.9 g of bead-milled CuHCF powder, using a vibrating device (Brennenstuhl Signograph 25, Tübingen, Germany). Subsequently, the other side of the tube was sealed off with additional glass wool and mounted into the instrument with two connectors. The packed column was then conditioned under N_2_ overnight under the following conditions to remove any remaining moisture and stabilize the detector: *T* = 423 K, *p* = 37.9 kPa, column flow = 1.5 mL/min, N_2_ inlet flow = 122.8 mL/min. Detector settings: H_2_ flow = 50 mL/min, air flow = 400 mL/min, N_2_ make-up flow = 50 mL/min. The column carrier gas flow was measured via an Agilent flow meter and adjusted to 1.5 mL/min. These settings were also used for the retention time acquisition of all probes at 423 K with a split ratio of 1:80. After each IGC experiment, the column was conditioned again overnight to cleanse it, allowing the baseline to stabilize. Methane (C_1_) was chosen to define the column hold-up time, *t*_M_ (in some studies referred to as *t*_0_), and measured prior to each experiment by injecting a known volume (25 µl) onto the column material to ensure its retention time remained constant.

### IGC free energy of adsorption

All measurements for deriving thermodynamic properties were performed with pure compounds, except for butane, and at infinite dilution. Care was taken that the peaks were symmetrical, and the probe retention times (*t*_R_) were independent of the injection volume. For the range methane to *n*-butane (C_1_, C_2_, *n*C_3_ and *n*C_4_), 25 µl of gas was injected into the IGC instrument. Volatiles *n*-pentane and *n*-hexane, *n*C_5_ and *n*C_6_, respectively, were injected as headspace, while the remaining hydrocarbons were injected as a liquid (0.1 µl). Vapour pressures were calculated via the Clausius Clapeyron equation. In between measurements with solvents, the solvent syringe was washed with acetone, flushed in a stream of N_2_ and dried to prevent any carry-over. For the selectivity experiments on the isomers of hexane, analogous settings and liquid injection volumes were utilized.

### IGC enthalpy of adsorption

Probes *n*C_4_-*n*C_7_ and toluene were injected onto CuHCF and measured at oven temperatures between 388 and 423 K in steps of 5 K. All measurements were performed in triplicate. Each time, 0.1 µl of liquid or 25 µl of headspace was injected into the IGC instrument and a split ratio of 1:80 was utilized. The column hold-up time *t*_M_ was measured prior to each injection of a new probe to calculate the corrected enthalpy values.

### IGC adsorption capacity & surface coverage

For the determination of adsorption isotherms, increased masses of the probe analytes were introduced. Split ratios for injected *n*C_5_–*n*C_7_ and toluene were 1:80 and 1:25 and the injection volumes varied between 0.02 and 2 µl. From the resulting tailing peaks the adsorption isotherms were calculated with the use of a modified version of the peak maximum (PM) method by Ho et al.^48^.

### Nitrogen sorption measurements for BET analysis

Nitrogen sorption measurements were carried out on a Microactive Tristar II Plus (Eindhoven, The Netherlands) 2.01 at 77 K. CuHCF powder was milled and dried before analysis and heated to 333 K and 423 K to compare the surface areas. The BET surface area was calculated from the adsorption data with Rouquerol criteria^49^. The fitting parameters were 0.6225 ± 0.3% and 0.0088 ± 0.1% for the slope and intercept, respectively.

## Results and discussion

### CuHCF powder characterization

Upon milling, which was performed to facilitate packing of the IGC column with CuHCF powder, the agglomerate particle size was reduced and made more homogeneous (Fig. S1 Supporting Information). BET analysis was performed to validate the milled CuHCF powder's integrity (Fig. S2), revealing a surface area of 7 m^2^/g for the heated powder used for packing the IGC column^47^. Prior to heating, the surface area was higher (375 m^2^/g), and the loss of water due to heating has most likely resulted in a lower surface area. The BET values are within the range reported for CuHCF (5–500 m^2^/g), but it is noted that the large variation in synthesis methods make a direct comparison difficult. The thermal stability of CuCHF was assessed by thermogravimetric analysis (Fig. S3), showing that the material is thermally stable up to at least 453 K after drying under an N_2_ atmosphere and is free of H_2_O after drying. Furthermore, under a certain set of experimental conditions the *t*_M_ slightly decreased from 0.63 to 0.5 min after 3 months of consecutive measurements and regeneration, indicating a high stability of the packed column material. The crystallinity and the presence of cubic lattices were confirmed by powder XRD (Fig. S4) and in accordance with reported CuHCF diffractograms^44^. Elevating the temperature of the CuHCF powder from room temperature to 423 K promoted a geometric transition from mono- to diclinic. The XPS data of the heated powder, as illustrated in Fig. S5, demonstrates a Cu:Fe ratio of 3:2. In addition, the Cu:Na ratio was found to be 3:1, evidencing that the majority of the sodium ions have been substituted by copper during the synthesis.

### IGC thermodynamic properties

#### Free energy of adsorption

**Figure 1:**
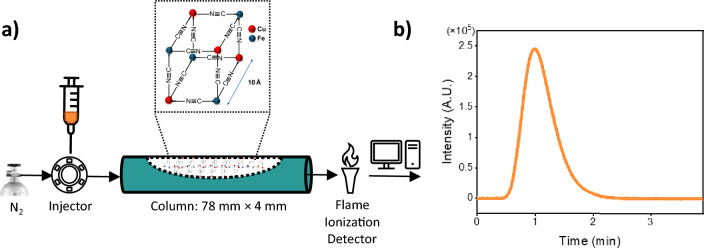
(**a**) Schematics of the IGC setup, including an inset displaying the unit cell and structure of CuHCF, (**b**) Chromatogram of *n*-hexane on CuHCF with *t*_R_ (retention time) = 1.0 min, *T* = 423 K.

IGC was employed for investigating the adsorption affinity of both linear and aromatic hydrocarbons towards CuHCF. A schematic of the utilized setup is given in Fig. 1a. The IGC comprises a standard GC instrument wherein the capillary column has been replaced by a short 4 mm i.d. glass tube containing milled CuHCF particles between glass wool plugs. A typical IGC chromatogram obtained during such measurements, in this experiment *n*-hexane (*n*C_6_), where the number in subscript denotes the total # of carbon atoms, is shown in Fig. 1b. Herein, the intensity on the vertical axis is related to the concentration of the probe in the gas-phase eluting from the column and the retention time (*t*_R_) on the horizontal axis relates to the interaction strength between the column material and the probe molecule. From Fig. 1b, it can be observed that *n*C_6_ elutes around *t*_R_ = 1.0 min and around 2.2 min it has fully eluted. Injections of increasing *n*C_6_ amounts, as well as those of other probes, allow calculating the adsorption isotherms (see “Adsorption capacity & surface coverage”).

**Figure 2:**
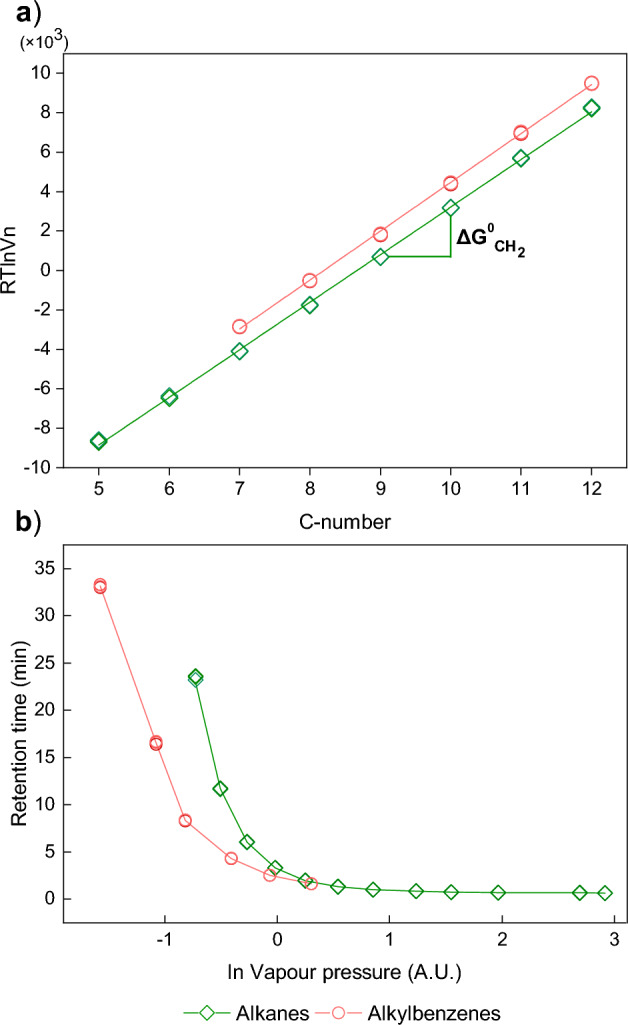
(**a**) Adsorption free energy calculation (*RT* ln*V*_n_ or Δ*G*_ads_) of *n*-alkanes (*n*C_5_–*n*C_12_) and alkylbenzenes (toluene up to *n*-hexylbenzene) according to the Dorris-Gray approach^50^. The graph depicts the average slope of triplicate injections, allowing for determination of the adsorption dispersive energy of a methylene group (${\Delta G^{0}_{\rm CH_{2}}}$, see added label in the graph). (**b**) Retention times of alkanes and alkylbenzenes plotted against their vapour pressures. The lines in the graph represent the average values derived from the triplicate measurements, serving as a visual guide for interpretation. Experiments were performed at 423 K.

The free energy of adsorption (Δ*G*_ads_), related to the isothermal adsorption per mole of molecules, was estimated by measuring the *t*_R_ values of additional *n*-alkanes (besides *n*C_6_) and alkylbenzenes, and further onward also more polar probes. The retention of these polar probes and alkylbenzenes involves both dispersive and specific components, compared to the sole dispersive component of *n*-alkanes, leading to higher absolute retention values ^48^. The retention difference then equates to Δ*G*_ads_, which can be used to predict the gas sensing properties of the material for different probes. The probes used were (1) C_1_, C_2_ and *n*C_3_ to *n*C_12_, and (2) alkylbenzenes toluene to *n*-hexylbenzene. The retention behavior of these two series on CuHCF is highlighted in Fig. 2. The Δ*G*_ads_ values have been calculated via the Dorris-Gray approach (Fig. 2a)^50^. For both series of probe molecules, Δ*G*_ads_ shows a positive linear correlation with increasing number of carbon atoms. This linear increase in Δ*G*_ads_ is due to London dispersion forces. As expected, the incremental ${\Delta G^{0}_{\rm CH_{2}}}$ values are equal for the alkylbenzenes and *n*-alkanes. The observed variation in retention volume between alkylbenzenes and alkanes could be a result of specific π–π interactions occurring between the aromatic rings and the π electrons within the C≡N bonds of CuHCF^51,52^. In addition, fundamental properties of the probes, such as vapour pressure and molecular shape and size, are factors that could contribute to probe-adsorbent interactions, e.g. through steric hindrance effects. In more detail, while a benzene ring experiences some steric hindrance due to its bulkiness, for a certain carbon number, the linear equivalent, being an alkane with a longer carbon chain, will have more steric hindrance.

To gain further insight into the effect of the vapour pressure on the retention time, Fig. 2b depicts the *t*_R_ values of the hydrocarbon probes against this parameter. Herein, alkanes C_1_, C_2_, *n*C_3_ and *n*C_4_ are nearly unretained and from *n*C_5_ onwards the retention times increase by roughly a factor of two per additional carbon atom. Interestingly, there is less retention for all alkylbenzenes on CuHCF compared to *n*-alkanes at equal vapour pressures. This implies a possible size-exclusion mechanism of the bulkier alkylbenzenes compared to linear *n*-alkanes, which is discussed further in “Hexane isomer selectivity”.

In addition to probing CuHCF with alkylbenzenes and *n*-alkanes, several polar compounds were also investigated as probes. Hence, similar IGC experiments were performed with two polar alkyl series: (1) alkylamines from methylamine (C_1_-NH_2_) to *n-*hexylamine (*n*C_6_-NH_2_), and (2) alcohols from methanol (C_1_-OH) to *n*-hexanol (*n*C_6_-OH). However, for both the linear amines and alcohols, none of the probe molecules eluted within the cut-off time (60 min), even when the temperature was increased to 180 °C. This indicates a very high affinity of these probes for CuHCF, suggesting that elution may only occur after a longer period. This high affinity is likely due to the strong-acid base characteristics of CuHCF, reported for other PBAs, stemming from interactions between the lone pairs of the O and N atoms with the metals of the lattice^40^. To shield the amine and hydroxyl groups from interacting with CuHCF, bulkier derivatives were also injected, namely triethylamine, aniline, dimethylaniline and 2-methylpropan-2-ol. For these bulkier analytes, only partial elution was seen within the cut-off time. The strong, but reversible interactions for the polar probes make CuHCF an interesting candidate for incorporation into sensing devices. CuHCF has already been investigated for its potential use in NH_3_ sensing and removal in farm poultry environments, exhibiting an adsorption capacity of 34 mg/g^42^. Yet, the integration and application of CuHCF in gas sensing devices is a field of study still in its early stages of development and stands to gain significant benefits from a comprehensive understanding of the underlying interactions involved.

#### Hexane isomer selectivity

**Figure 3:**
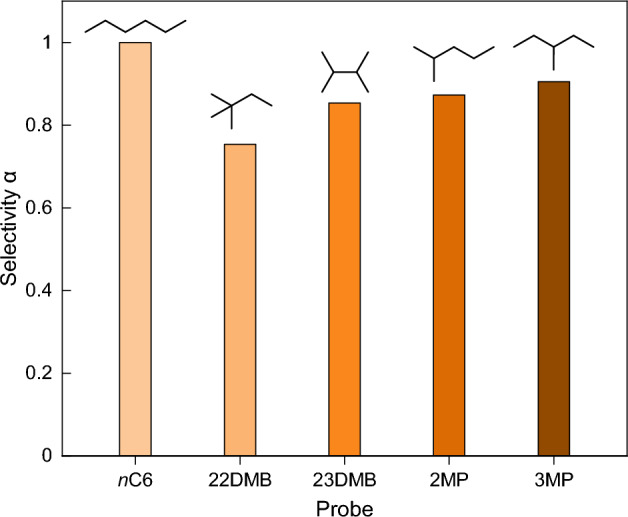
IGC-based selectivity values of the isomers of hexane on CuHCF. Probe abbreviations: 2MP (2-methylpentane), 3MP (3-methylpentane), 22DMB (2,2 dimethylbutane), 23DMB (2,3 dimethylbutane). The selectivity is expressed versus *n*-hexane (*n*C_6_) and experiments were performed at *T* = 423 K.

The possible size-exclusion effect observed for alkylbenzenes (“Free energy of adsorption”) inspired the study of other branched alkanes on CuHCF, namely *n*C_6_ and its four isomers. Quantifying the adsorption strength of branched C_6_ isomers on porous materials holds significant importance for industrial applications related to gas monitoring, particularly for optimizing process conditions, such as in fuel production. There is an increasing need to develop porous materials that can selectively detect and separate *n*C_6_ from its isomers, due to their relatively similar boiling points, chemical inertness, and non-polar nature^53–55^. The selectivity results of branched C_6_ isomers injected onto CuHCF are shown in Fig. 3. Herein, hexanes 2MP (2-methylpentane), 3MP (3-methylpentane), 22DMB (2,2 dimethylbutane), 23DMB (2,3 dimethylbutane) and *n*C_6_ were injected separately onto the CuHCF-packed IGC column. Here, the selectivity (*α*) is defined as the ratio of the net retention times *versus* the one of *n*-hexane, where the net retention time is *t*_R_–*t*_M_. The results show that the bulkiest hexanes 22DMB and 23DMB indeed have a lower *α* (0.76, 0.82, respectively) than 2MP and 3MP (0.83, 0.86, respectively). Furthermore, all four isomers have a lower retention on CuHCF compared to *n*C_6_. These results indicate that branched hexane isomers are less compatible with the pores of CuHCF due to the shape and minor boiling point differences, resulting in a lower selectivity compared to *n*C_6_. This finding illustrates the possibility of utilizing this PBA for selective gas sensing purposes. It is realized that the observed selectivity is not particularly high, i.e., 0.73 < *α* < 1 rather than *α* ≪ 1. However, it should be noted that a relatively short column was used in this study and interestingly, it was sufficient to observe differences in the retention times of the hexane isomers for obtaining the related thermodynamical properties. While IGC is primarily focused on characterizing column material properties rather than separation, achieving longer columns and thereby enhancing selectivity, as demonstrated by Yusuf et al*.*^33^, could potentially improve separation efficiency when analysing complex mixtures. Nevertheless, the feasibility of extending the column length beyond the current setup's limitations (due to an inflexible glass column and small oven size) is challenging.

#### Enthalpy of adsorption

The adsorption affinity of hydrocarbons towards CuHCF has been established through Δ*G*_ads_ estimations and the hexane isomer selectivity experiments. Another crucial parameter that can be determined via IGC is the enthalpy of adsorption (Δ*H*_ads_), which represents the energy necessary for binding the probe to the adsorbent. It is derived from the general Arrhenius equation for studying reaction kinetics at a range of temperatures:

1 $$k=A{e}^{\frac{-\Delta H\text{ads}}{RT}}$$

where *k* is the retention factor, *A* the pre-exponential factor, *R* the ideal gas constant (8.314 J/mol/K) and *T* the temperature (K). The slope value, extrapolated from the exponential fit of the Arrhenius plot, is used to calculate Δ*H*_ads_:

2 $$\text{Slope }= \frac{-\Delta H\text{ads}}{R}$$

**Figure 4:**
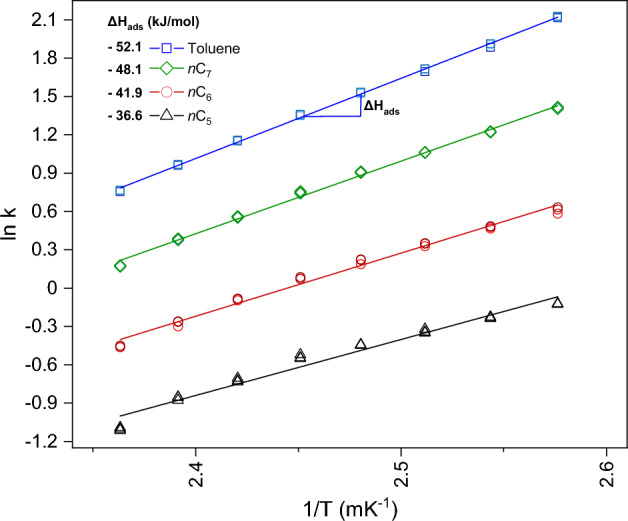
Arrhenius relationship (Eq. 2) for alkanes *n*C_5_–*n*C_7_ and toluene injected on CuHCF. From the average slope depicted in the graph for each probe injection conducted in triplicate, the Δ*H*_ads_ values have been calculated according to Eq. (2). Experiments were performed within the range of *T* = 388–423 K.

In Fig. 4, the Arrhenius relationship for the probes *n*C_5_-*n*C_7_ and toluene is visualized. For each hydrocarbon, a linear trend is observed. As expected, the resulting Δ*H*_ads_ values increase upon increasing alkyl length, which can be rationalized by the additive nature of the dispersive interactions. The Δ*H*_ads_ difference between toluene and *n*C_7_ is 4 kJ/mol, suggesting a modest aromatic carbon contribution from the benzene group. Given the variations in parameters and material compositions across studies, conducting a detailed comparison with Autie-Castro^56^ and Yusuf et al.^34,56^, such as their use of MOFs for enthalpy evaluation through IGC, is practically not achievable. However, despite these differences, their findings illustrate that the trends in alkyl adsorption energies remain comparable, with all values falling within a similar range. For instance, in their investigation of the well-studied porous framework material copper benzene-1,3,5-tricarboxylate (Cu-BTC), Autie-Castro et al. and Yusuf et al. reported Δ*H*_ads_ values for *n*C_5_, *n*C_6_, and *n*C_7_ ranging from approximately − 57.0 to − 70.2 kJ/mol. Similarly, toluene exhibited an adsorption energy of around − 66.5 kJ/mol. Additionally, in our study, it was found that for CuHCF, the increment per methylene group was on average 5.8 kJ/mol, a value in between those of Cu-BTC and Fe-BTC (approximately 6.6 and 1.3 kJ/mol/CH_2_, respectively)^56^. This indicates a consistent pattern across different materials, despite the specific differences in experimental conditions and methodologies used in the studies.

#### Adsorption capacity & surface coverage

**Figure 5:**
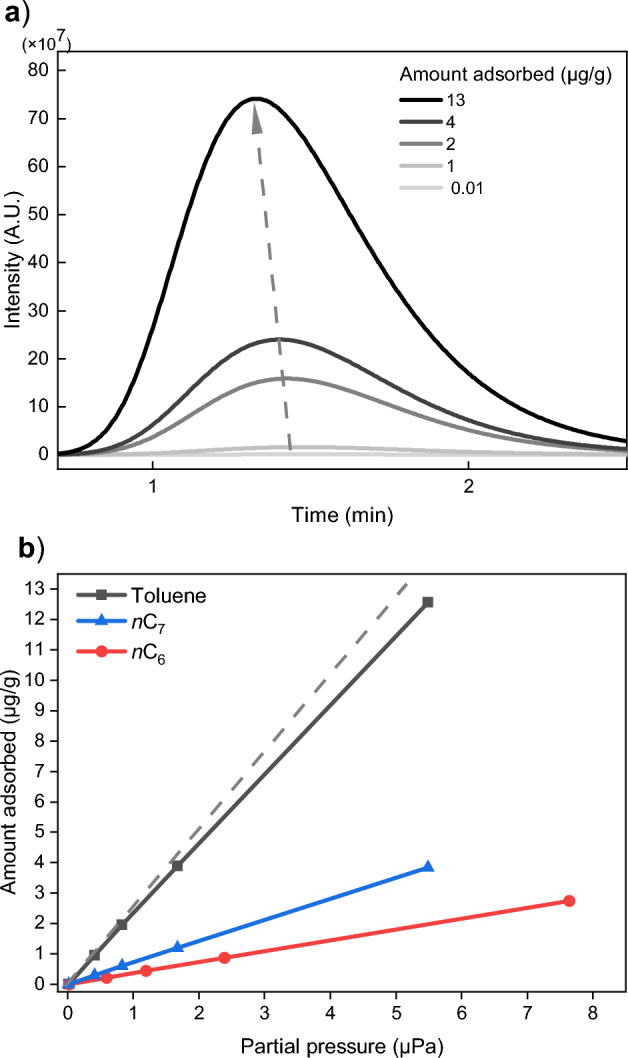
(**a**) Chromatograms of increasing toluene volume injections into a CuHCF IGC column. The dashed arrow indicates the shift in *t*_R_ due to oversaturating the column material; and (**b**) Calculated adsorption isotherms from figure (**a**) according to the PM method described by Ho et al.^48^. The extrapolated dashed line indicates the non-linear behavior for toluene due to oversaturation. Experiments were conducted at *T* = 423 K.

In addition to investigating adsorption parameters, such as Δ*G*_ads_ and Δ*H*_ads_, IGC can also serve as a valuable tool for estimating adsorption capacity and surface coverage. These properties are obtained by injecting increased amounts of probe and calculating the surface coverage from the adsorption isotherm, according to the Peak Maximum method^48^. Herein, the partial pressures obtained from the retention volumes and the injected probe mass are plotted and the result is integrated to obtain the amount adsorbed for each partial pressure. The adsorption capacities of the probes *n*C_6_, *n*C_7_ and toluene onto CuHCF are shown in Fig. 5. Upon increasing the injected toluene volume, the *t*_R_ value becomes smaller (Fig. 5a), which can be rationalized by the effect of oversaturating the CuHCF column material. For *n*C_6_ no shift in *t*_R_ was observed (Fig. S6a) and for *n*C_7_ the *t*_R_ shift was only minor (Fig. S6b), and this is also noticeable in the shape of the adsorption isotherms (Fig. 5b), *i.e.* their flattening. The adsorption isotherms were calculated from the *t*_R_ shifts using Eq. (3)^48^

3 $${n}_{\text{ad}}= \frac{1}{{m}_{\text{s}}}\cdot \int \frac{{V}_{\text{N}}}{RT} dP$$

here, *n*_ad_ stands for the absorbed amount per g of solid; *R* is the ideal gas constant and *T* the temperature of the oven in *K*; and *m*_s_ the mass of the adsorbent. For each probe, a linear trend is found with a slight flattening for toluene and *n*C_7_ from 0.5 µg/g onwards (Fig. 5b). The latter suggests that oversaturation of the column starts to occur when exceeding 0.5 µg/g. For *n*C_6_ and shorter *n*-alkanes, *t*_R_ remains constant within this concentration range. For all these probes, it is evident that the saturation point has not been reached, indicating that the maximum adsorption capacity of CuHCF is larger than the mass range studied here. In the context of gas sensing applications, this wide linear regime is advantageous, as ideally the gas concentration gives a linear sensor response. To calculate the theoretical maximum surface coverage that can be achieved for each probe, Eq. (4) was utilized^48^:

4 $${n}_{m}= \frac{\sigma }{{a}_{m}\cdot {N}_{a}}$$

here, *σ* is the specific surface area in m^2^/g, *a*_m_ the surface area occupied by a probe molecule in m^2^/molecule, calculated via the density of the probe in bulk liquid state^57^, *N*_a_ Avogadro’s number in molecules/mole, and *n*_m_ the monolayer capacity in molecules per g of column material. For toluene, at the maximum injected amount in this experiment, 46.4% of the total surface was covered, and for* n*C_7_ and *n*C_6_ this was 16.8% and 13.8%, respectively.

## Conclusions

This study provides insights in the gas sensing properties of CuHCF towards a broad range of probes with the use of IGC and demonstrates the utility of IGC for obtaining quantitative data on these properties. We have observed that the adsorption for alkanes was higher on this material compared to alkylbenzenes due to steric effects, vapour pressure, and π–π interactions. Furthermore, the pore accessibility is higher for alkanes, which was also noticeable when studying the adsorption enthalpy and selectivity of the five isomers of hexane. This exclusion effect makes CuHCF an attractive candidate for its incorporation into affinity coatings of gas sensors. Polar probes like amines and alcohols interact strongly with CuHCF, which is likely attributed to the acid–base interactions, which are absent for the alkane and alkylbenzene series. Such strong interactions are particularly intriguing for applications where selectivity is crucial, such as in affinity coatings and advanced high-adsorption materials. Additionally, the adsorption capacity for the injected masses of *n*C_6_, *n*C_7_ and toluene was still in the linear regime, which is advantageous for sensing purposes.

To further assess the adsorption contribution of the transition metals present in the lattice, a wider range of Prussian Blue Analogues can be studied in the future. We successfully demonstrated the potential of IGC to study the gas adsorption properties of these materials. To further improve this potential, studies towards RT conditions can also be conducted, although side effects such as diffusion should be minimized. The use of IGC to quantify the surface-dependent adsorption behavior of volatile organic probes on PBAs—and porous nanomaterials in general—provides a facile way to obtain valuable insights for their use in gas sensing devices. We consider IGC to be particularly useful in the screening of such materials, before their implementation in sensor coatings, gas separation and gas capturing applications.

### Supplementary Information

Supplementary Figures.

## Data Availability

The data that support the findings of this study are available from the corresponding author on request.
