# Supplementary material for: Exploring affinity between organic probes and Prussian Blue Analogues via inverse gas chromatography
Source: Sci Rep. 2024 Jun 17;14:13904. doi: 10.1038/s41598-024-62939-7 (PMC11183049; doi:10.1038/s41598-024-62939-7)
Supplement: Supplementary file 1 — Supplementary Figures. [file 41598_2024_62939_MOESM1_ESM.pdf]

Exploring Affinity between Organic Probes and Prussian Blue Analogues via Inverse Gas Chromatography

Stijn Paulusma[1], Kaustub Singh[2,5], Tom Smeding[1], Jayaruwan G. Gamaethiralalage[1,3], Frank W. Claassen[1], Hans Beijleveld[1], Hans-Gerd Janssen[1,4*], Louis C.P.M. de Smet[1*]

[1]Laboratory of Organic Chemistry, Wageningen University & Research, Stippeneng 4, 6708 WE, Wageningen, The Netherlands

[2]Department of Chemical Engineering, Delft University of Technology, Van der Maasweg 9, 2628 CN, Delft, The Netherlands

[3]Section of Chemical Science and Engineering, Department of Chemistry and Bioscience, Aalborg University, Niels Bohrs Vej 8, 6700, Esbjerg, Denmark

[4]Unilever Foods Innovation Centre — Hive, Bronland 14, 6708 WH, Wageningen, The Netherlands

[5]FrieslandCampina Innovative Centre, Bronland 20, 6708 WH, Wageningen, The Netherlands.

* Corresponding author: Louis C.P.M. de Smet

Table of Contents

Figure S1: SEM image 2

Figure S2: BET linearization plot 2

Figure S3: TGA plot 3

Figure S4: X-ray diffractogram 3

Figure S5: XPS data 4

Figure S6: Chromatograms adsorption capacities 5

Figure S7: Photos of the IGC equipment 6

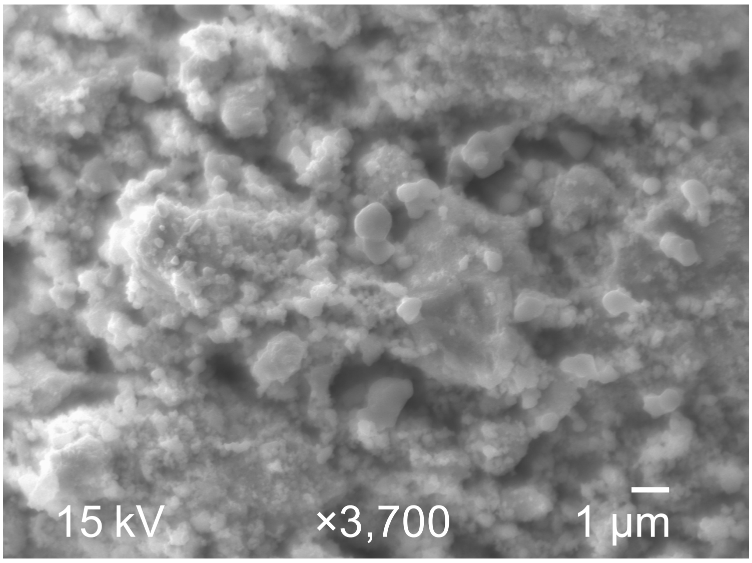

Figure S1: SEM image of the milled CuHCF material used for IGC measurements at 3700× magnification.

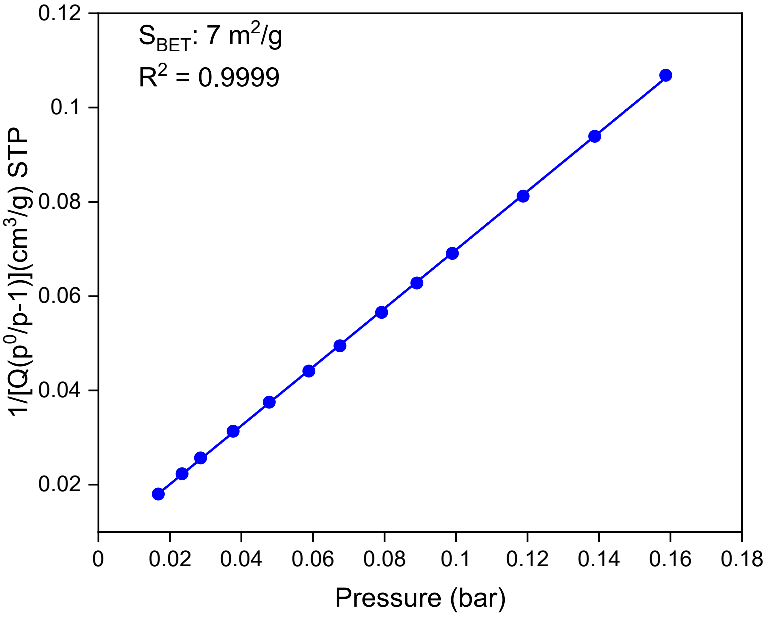

Figure S2: BET linearization of the milled CuHCF material heated at T = 423 K.

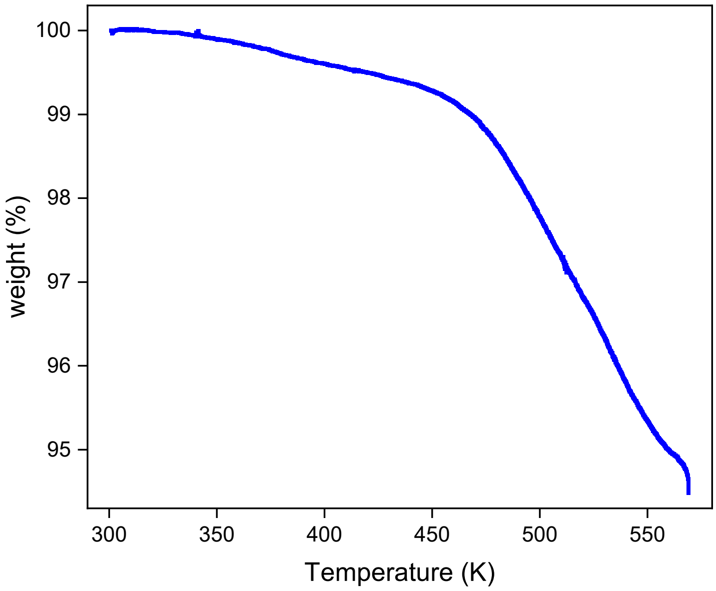

Figure S3: TGA plot of the milled CuHCF material used for IGC experiments. The CuHCF powder was dried at T = 373 K to ensure no moisture was present.

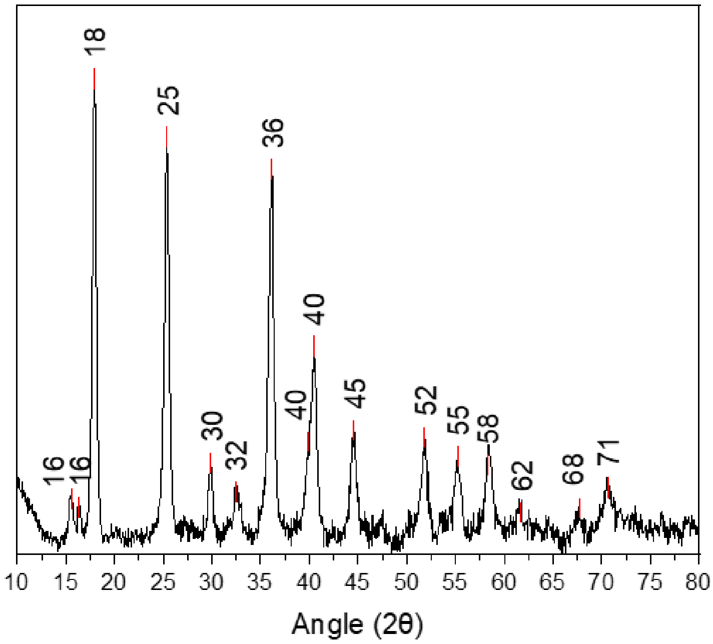

Figure S4: X-ray diffractogram of the milled CuHCF material used for conducting IGC experiments.

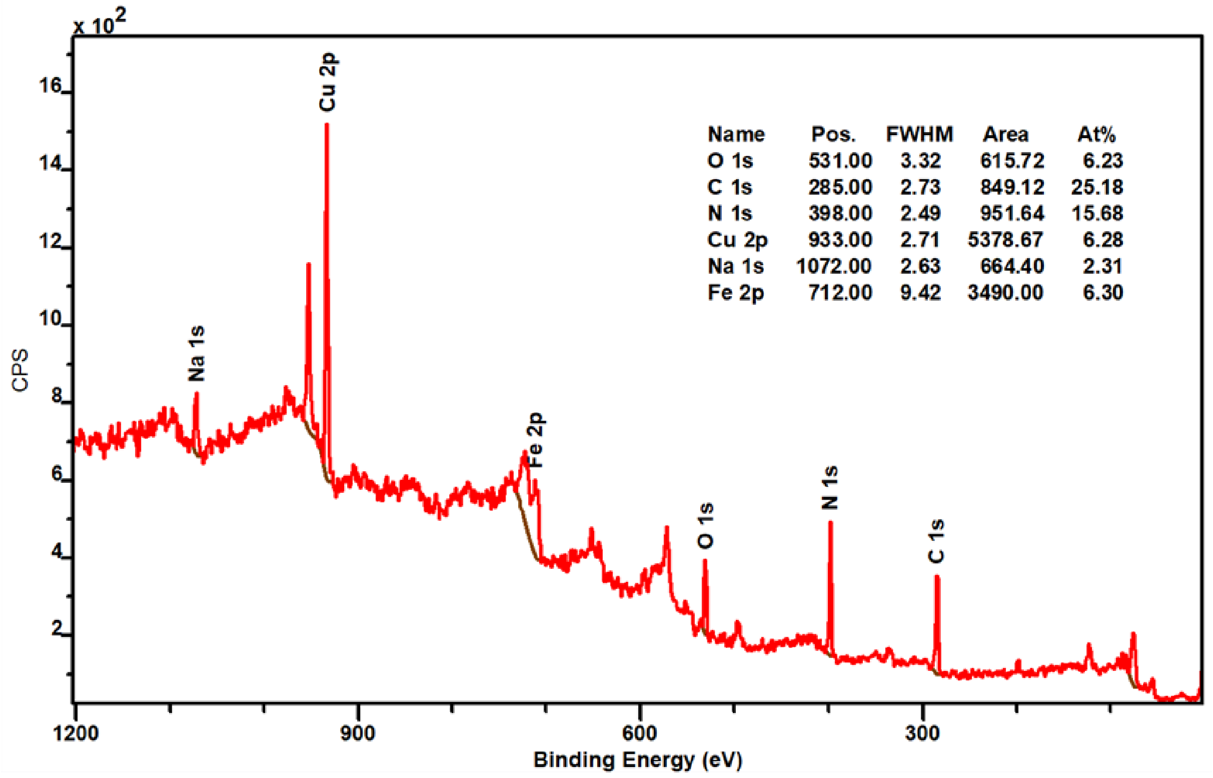

Figure S5: XPS spectrum of the milled CuHCF material used for IGC experiments.

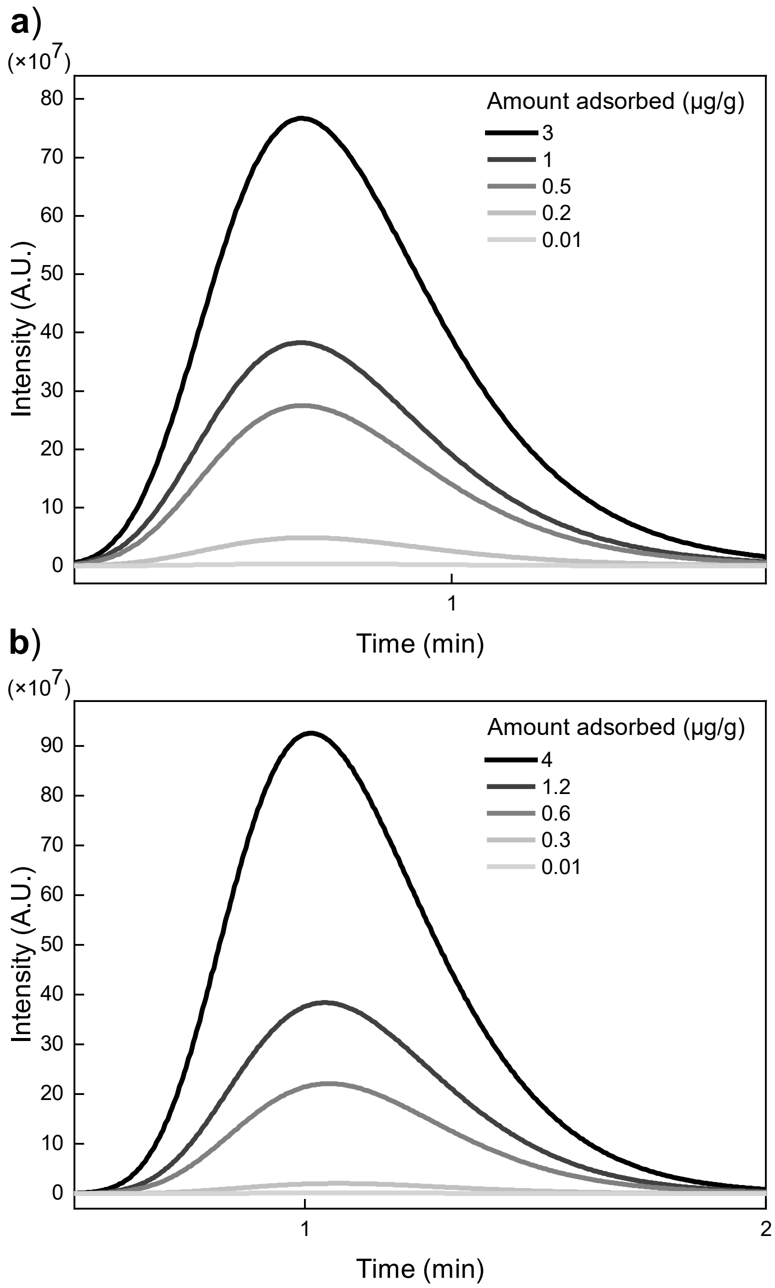

Figure S6: a) Chromatograms of increasing *n*-hexane (nC_6) volume injections onto a CuHCF-packed IGC column **b)** Chromatograms of increasing *n*-heptane (nC_7) volume injections onto a CuHCF-packed IGC column.

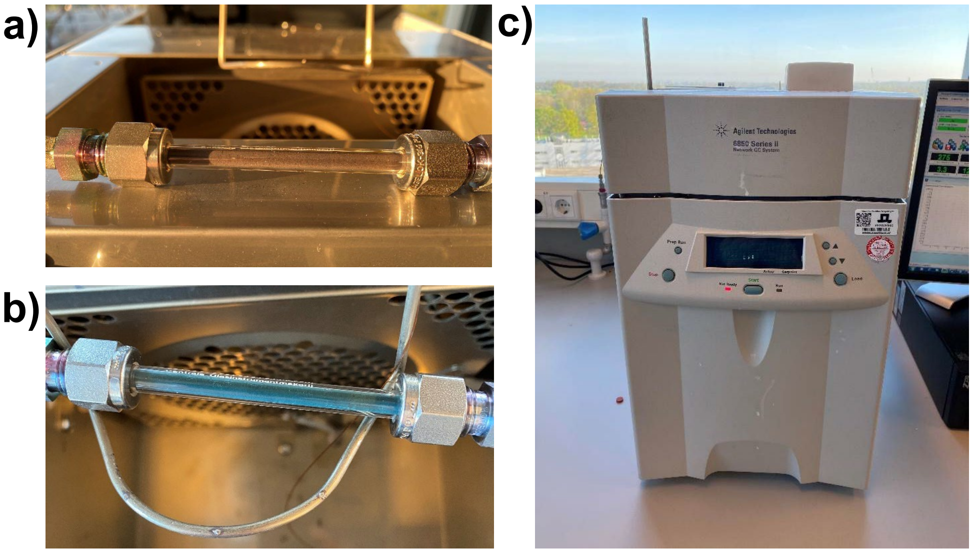

Figure S7: Photos of the IGC equipment. **a)** Packed CuHCF column before conditioning overnight at 423 K, **b)** CuHCF column after conditioning. **c)** Modified Agilent 6850 series II Gas chromatograph.
